# Supplementary material for: Tracking carrier protein motions with Raman spectroscopy
Source: Nat Commun. 2019 May 20;10:2227. doi: 10.1038/s41467-019-10184-2 (PMC6527581; doi:10.1038/s41467-019-10184-2)
Supplement: Supplementary file 1 — Supplementary Information [file 41467_2019_10184_MOESM1_ESM.pdf]

Supplementary Information for
Tracking carrier protein motions with Raman spectroscopy

Epstein et al.

Index of Supplementary Figures and Tables

Supplementary Figures

Supplementary Figure 1: The acyl carrier protein as a central hub of the synthase
Supplementary Figure 2: Intrinsic measurement timescale from simulated Raman spectra
Supplementary Figure 3: Raman spectrum of WhiE ACP loaded with 4-pentynoic acid.
Supplementary Figure 4: Sequence of DNA insert for Rat ACP plasmid
Supplementary Figure 5: ^{1}H NMR spectrum of 4-pentynoic acid (C5 probe)
Supplementary Figure 6: ^{1}H NMR spectrum of 7-octynoic acid (C8 probe)
Supplementary Figure 7: ^{1}H NMR spectrum of 12-tridecynoic acid (C13 probe)
Supplementary Figure 8: LC trace of *holo*-EcACP
Supplementary Figure 9: ES mass spectrum of *holo*-EcACP
Supplementary Figure 10: ES mass spectrum of *acyl*-EcACP C5
Supplementary Figure 11: ES mass spectrum of *acyl*-EcACP C8
Supplementary Figure 12: ES mass spectrum of *acyl*-EcACP C13
Supplementary Figure 13: ES mass spectrum of *holo*-Act ACP
Supplementary Figure 14: ES mass spectrum of *acyl*-Act ACP C8
Supplementary Figure 15: ES mass spectrum of *holo*-Rat ACP
Supplementary Figure 16: ES mass spectrum of *acyl*-Rat ACP C8
Supplementary Figure 17: ES mass spectrum of *holo*-Arm ACP
Supplementary Figure 18: ES mass spectrum of *acyl*-Arm ACP C8
Supplementary Figure 19: ES mass spectrum of *holo*-Ben ACP
Supplementary Figure 20: ES mass spectrum of *acyl*-Ben ACP C8
Supplementary Figure 21: ES mass spectrum of *holo*-WhiE ACP
Supplementary Figure 22: ES mass spectrum of *acyl*-WhiE ACP C5
Supplementary Figure 23: ES mass spectrum of *acyl*-WhiE ACP C8
Supplementary Figure 24: LC trace from *acyl*-Act ACP C8 spiked with free probe
Supplementary Figure 25: ES mass spectrum of 7-octynoic acid (C8 probe)
Supplementary Figure 26: ES mass spectrum of *acyl*-Act ACP C8
Supplementary Figure 27: SDS PAGE of *holo*- and *acyl*-ACPs
Supplementary Figure 28: Urea PAGE of *holo*- and *acyl*-ACPs
Supplementary Figure 29: CD spectra of EcACP with alkyne-labeled substrates
Supplementary Figure 30: CD spectra of Rat ACP with an alkyne-labeled substrate

Supplementary Tables

Supplementary Table 1: Mode, mean, and FWHM of alkyne probe Raman peak

Supplementary References

Supplementary Figures

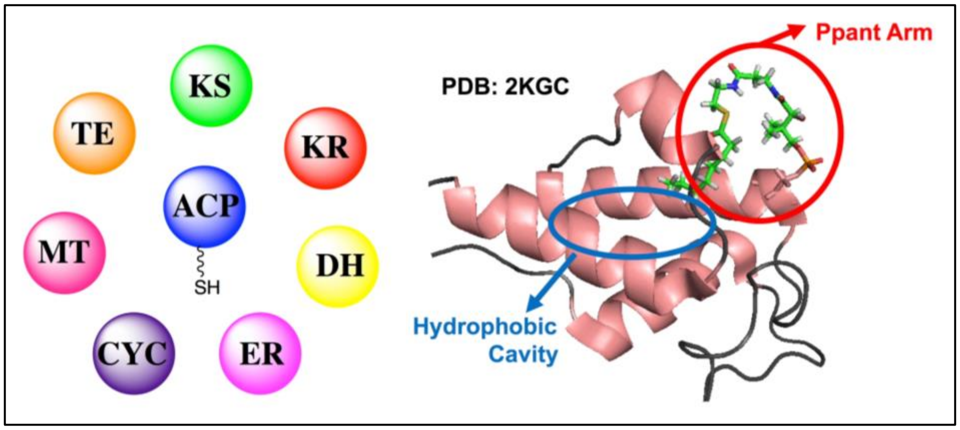

Supplementary Figure 1: The acyl carrier protein as a central hub of the synthase. The core domains of the *E. coli* fatty acid synthase and type II polyketide synthase (left) include the acyl carrier protein (ACP), keto synthase (KS), keto reductase (KR), dehydratase (DH), enoyl reductase (ER), cyclase (CYC), malonyl transferase (MT), and the thioesterase (TE). An ACP is central to each synthase, with the role of shuttling the growing compound among the various catalytic domains. ACPs (structure of octanoyl-Act ACP at right, PDB: 2KGC) feature a Ppant arm (inside red circle), which covalently tethers all intermediates as thioesters and allows the ACP to sequester molecular cargo within its hydrophobic cavity (blue circle).

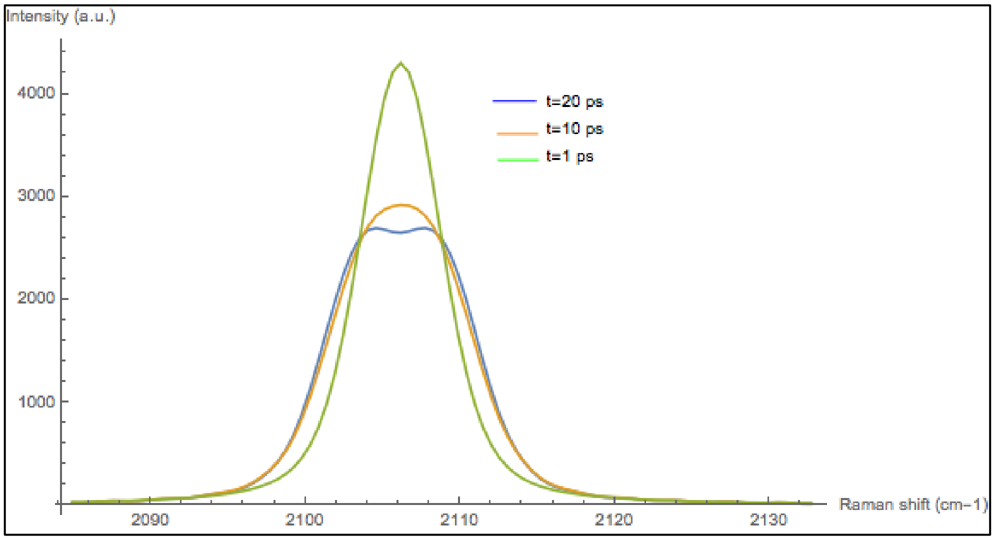

Supplementary Figure 2: Intrinsic measurement timescale from simulated Raman spectra. The simulated lineshapes above assume two spectral subpopulations centered at 2105 and 2110 cm^{-1}, in the frequency region of the observed alkyne stretching peaks, with a vibrational lifetime (total dephasing) of 5 ps and a Gaussian FWHM of 10 cm^{-1} (which roughly matches the spectrum of the probes in aqueous buffer), in dynamic exchange with each other at varying exchange times (*t* here is 1/exchange rate). The standard spectral lineshape treatment of Kubo and Anderson was adopted for this calculation.[1,2] Below 20 ps the lineshape is invariant and the two spectral subpopulations are distinguishable as both contributing to the lineshape, which is clearly broader than 10 cm^{-1}. At an exchange time of 10 ps, some minor coalescence due to exchange is observed, and at *t*=1 ps, the band has narrowed and the two populations are becoming indistinguishable. This simulation demonstrates that the intrinsic timescale of this particular measurement for distinguishing between probe environments is 20 ps or slower, or generally on the tens of ps timescale with some variation that depends on the exact separation between spectral components. All structures with dynamics that happen on timescales slower than 20 ps will be resolved in the probe Raman spectrum. (This is a general advantage of all vibrational probes as compared to, for example, NMR measurements: the fast frequencies associated with molecular vibrations lead to very short intrinsic timescales and resolution of conformational probe distributions in the spectrum).

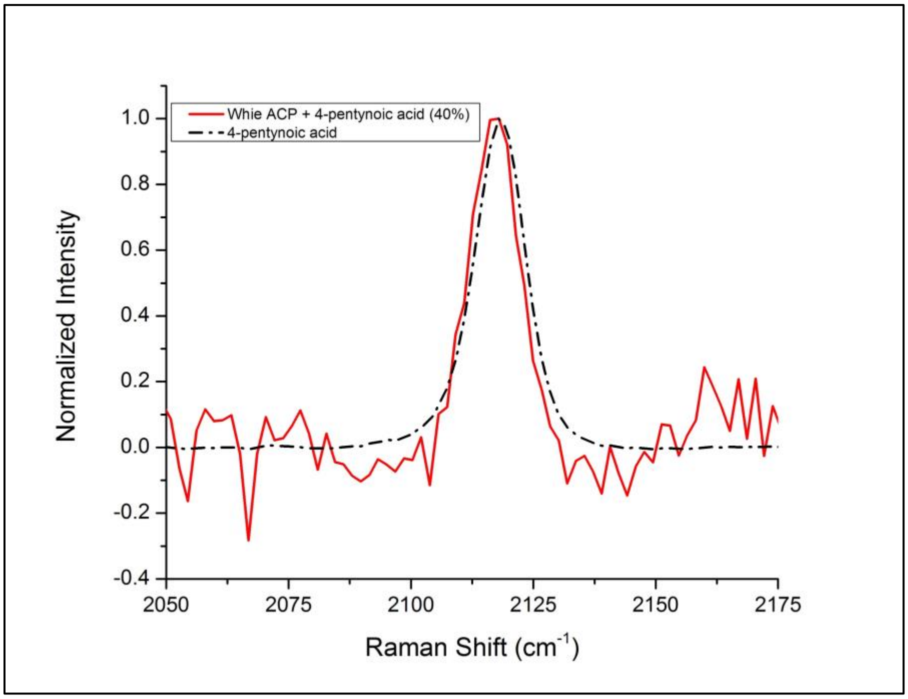

Supplementary Figure 3: Raman spectrum of WhiE ACP loaded with 4-pentynoic acid. The Raman spectrum indicates that WhiE ACP (in 50 mM sodium phosphate buffer pH 7.6) does not sequester the C5 probe (4-pentynoic acid), due to a lack of a peak shift relative to the signal of the isolated probe in aqueous solution. LC-MS data (Supplementary Figure 22) indicate that only an estimated 40% of the ACP sample was converted from the *holo* to the *acyl* form, but this non-quantitative probe labeling is not expected to interfere with data collection because the untagged *holo*-ACP does not provide a signal in this spectral region. Source data are provided as a Source Data file.

Rat ACP DNA Insert Sequence:
ggggaattgtgagcggataacaattcccctctagaaataattttgtttaactttaagaaggagatataccatgggcagcagccatcatca
*tcatcatcacagcagcggcctggtgccgcgcggcagcCATATG*ggtgatggtgaagcccagagggatctggtgaaagcagtggca
cacatcctaggcatccgcgacctcgcagggattaacctggacagctcgctggcagacctcggcctggactcgctcatgggtgtggaagtg
cgccagatcctggaacgtgaacatgatctggtgctacccattcgtgaagtacggcaactcacactgcggaagcttcaggaaatgtcctccaa
ggctggctcagacactgagttggcagcccccaagtccaagaattga*GAATTCgagctccgtcgacaagcttgcggccgcactcgag*
caccaccaccaccaccactgagatccggctgctaacaaagcccgaaaggaagc

Supplementary Figure 4: Sequence of DNA insert for Rat ACP plasmid. The nucleotide sequence of the DNA insert ordered to create the Rat ACP plasmid is shown above. Overlap with the pET28 vector (*italics*) and the restriction sites (CAPS) are indicated as well.

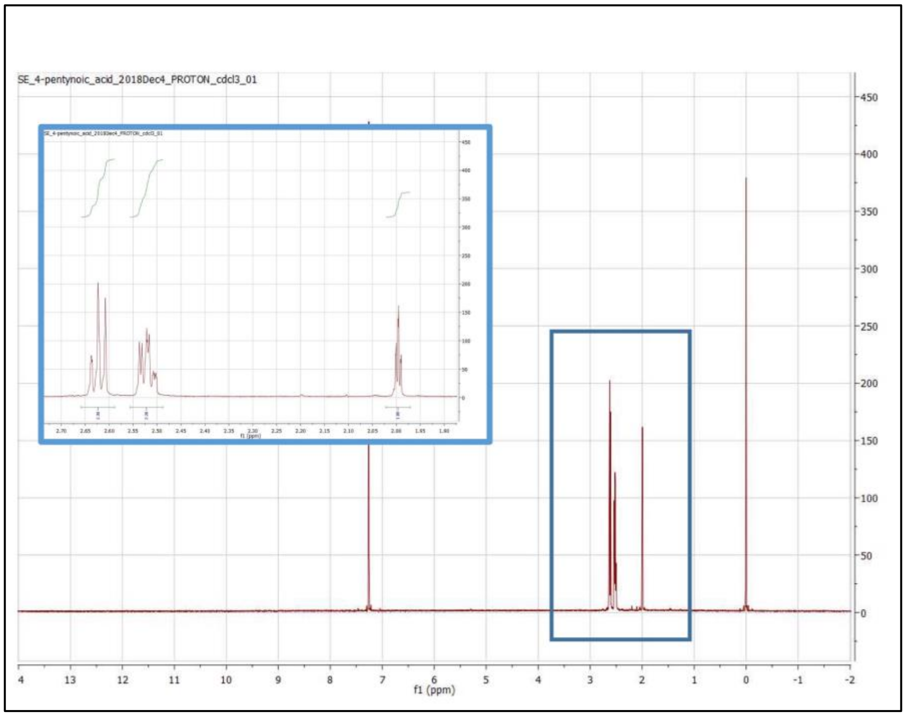

Supplementary Figure 5: ^{1}H NMR spectrum of 4-pentynoic acid (C5 probe).
All observed NMR peaks correspond to pure 4-pentynoic acid. ^{1}H NMR($CDCl_3$): δ 2.00 (td, 1H, CH, J=2.7 Hz, 0.7 Hz), δ 2.52 (td, 2H, CH_2, J=7.3 Hz, 2.7 Hz), δ 2.62 (t, 2H, CH_2, J=7.1 Hz). The ^{1}H NMR spectrum was acquired using a Varian 500 MHz NMR spectrometer to verify the purity of the commercially obtained alkyne-containing carboxylic acids. Deuterated solvent $CDCl_3$ is used. Source data are available from Open Science Framework (DOI 10.17605/OSF.IO/RKD4E).

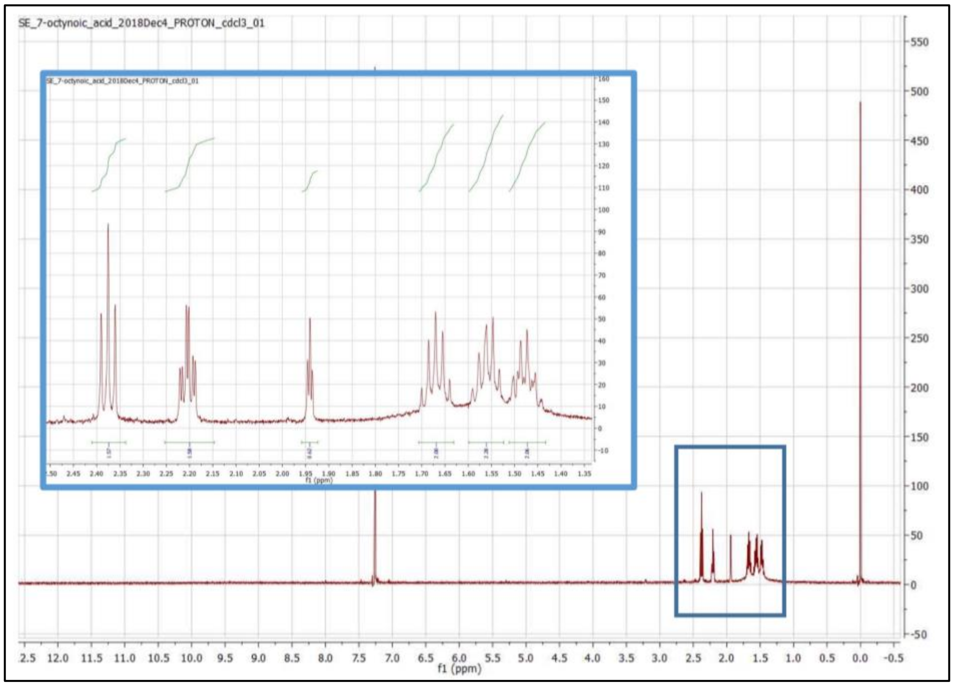

Supplementary Figure 6: ^{1}H NMR spectrum of 7-octynoic acid (C8 probe).
The observed NMR peaks correspond to pure 7-octynoic acid. ^{1}H NMR($CDCl_3$): δ 1.48 (m, 2H, CH_2), δ 1.56 (quint, 2H, CH_2, J=7.2Hz), δ 1.67 (quint, 2H, CH_2, J=7.5 Hz), δ 1.95 (td, 1H, CH, J=2.7 Hz, 0.7 Hz), δ 2.20 (td, 2H, CH_2, J=7.0 Hz, 2.6 Hz), δ 2.38 (t, 2H, CH_2, J=7.6 Hz). The ^{1}H NMR spectrum was acquired using a Varian 500 MHz NMR spectrometer to verify the purity of the commercially obtained alkyne-containing carboxylic acids. Deuterated solvent $CDCl_3$ is used. Source data are available from Open Science Framework (DOI 10.17605/OSF.IO/RKD4E).

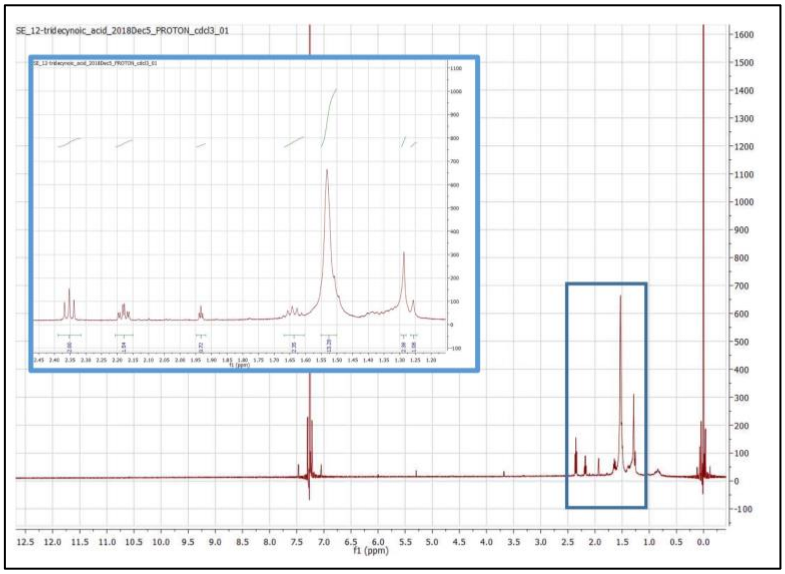

Supplementary Figure 7: ^{1}H NMR spectrum of 12-tridecynoic acid (C13 probe).
The observed NMR peaks correspond to pure 12-tridecynoic acid. ^{1}H NMR($CDCl_3$): δ 1.26 (br, 1H, CH_2), δ 1.29 (br, 2H, CH_2), δ 1.53 (br, 13H, CH_2),1.63 (quint, 2H, CH_2, J=7.3 Hz), δ 1.93 (td, 1H, CH, J=2.8 Hz, 0.5 Hz), δ 2.18 (td, 2H, CH_2, J=7.1 Hz, 2.7 Hz), δ 2.35 (t, 2H, CH_2, J=7.5 Hz). The ^{1}H NMR spectrum was acquired using a Varian 500 MHz NMR spectrometer to verify the purity of the commercially obtained alkyne-containing carboxylic acids. Deuterated solvent $CDCl_3$ is used. Source data are available from Open Science Framework (DOI 10.17605/OSF.IO/RKD4E).

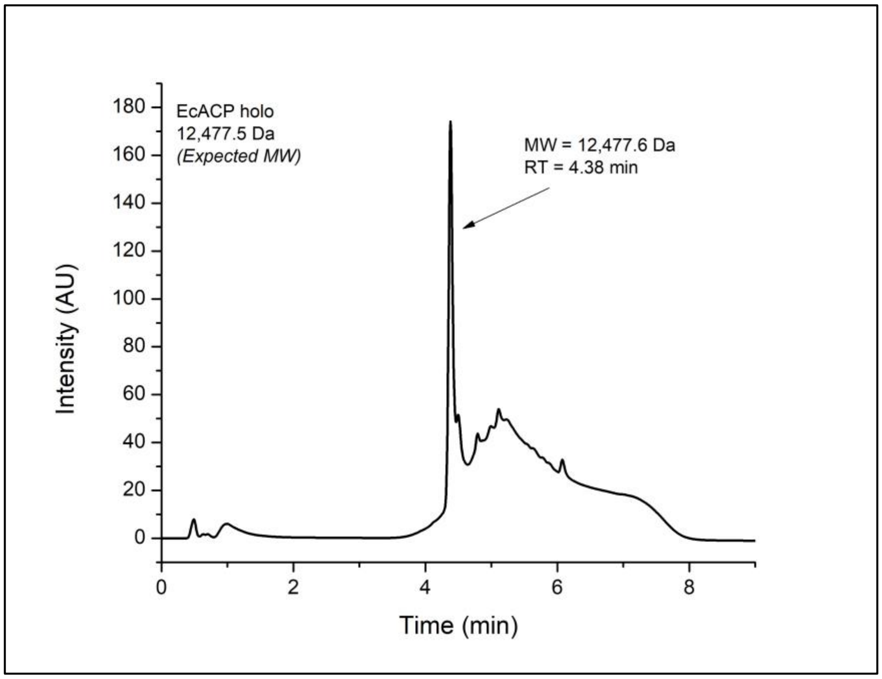

Supplementary Figure 8: LC trace of *holo*-EcACP .
The LC trace (measured at 254 nm) observed over the course of the LC-MS method is shown for *holo*-EcACP. The same general elution profile was observed for each ACP sample.

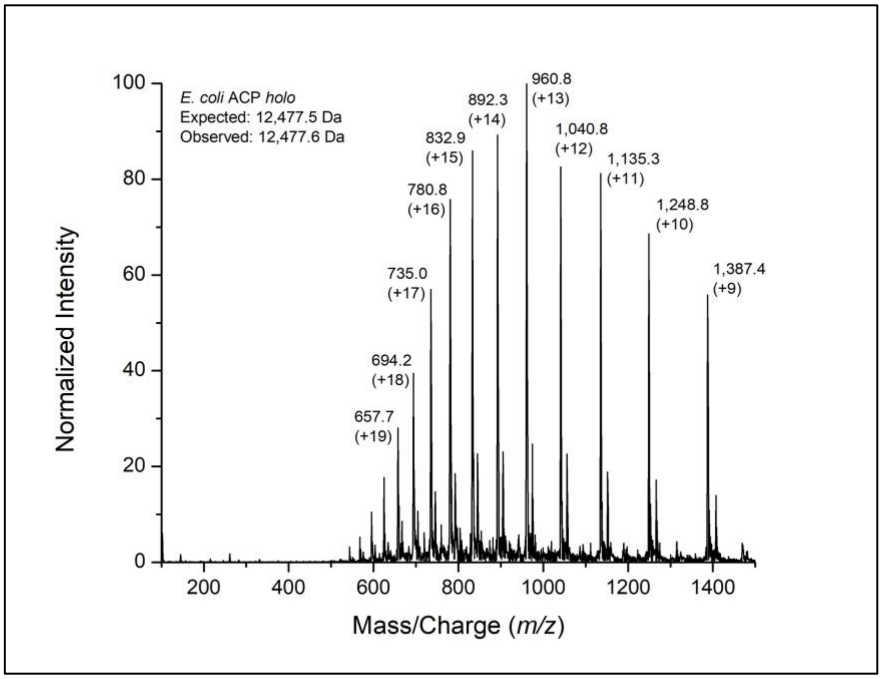

Supplementary Figure 9: ES mass spectrum of *holo*-EcACP.
The different charge (z) states are marked on the plot and are calculated according to the theoretical molecular weight of the ACP (minus the N-terminal methionine).

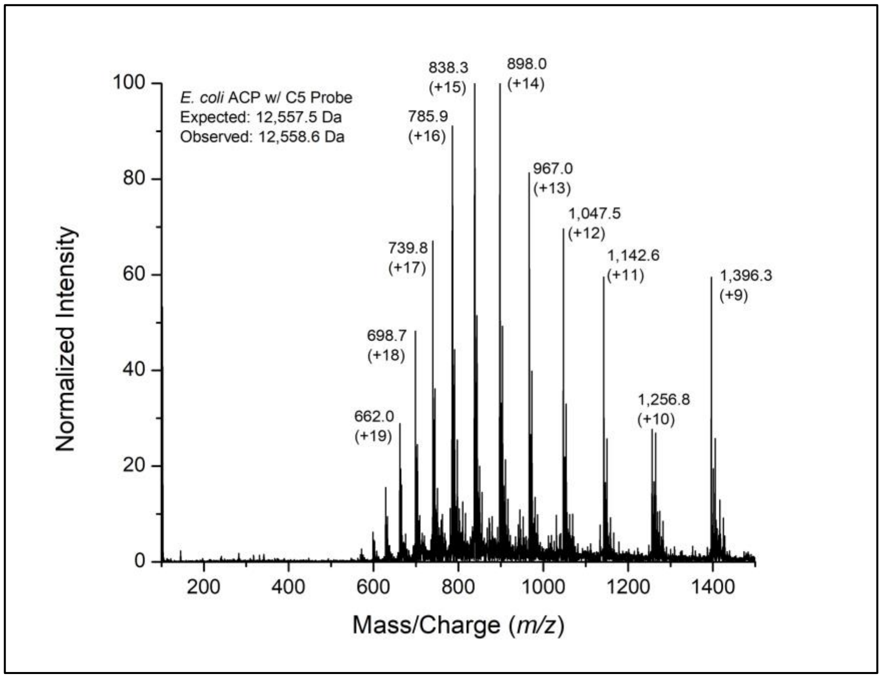

Supplementary Figure 10: ES mass spectrum of *acyl*-EcACP C5.
The different charge (z) states are marked on the plot and are calculated according to the theoretical molecular weight of the ACP (minus the N-terminal methionine).

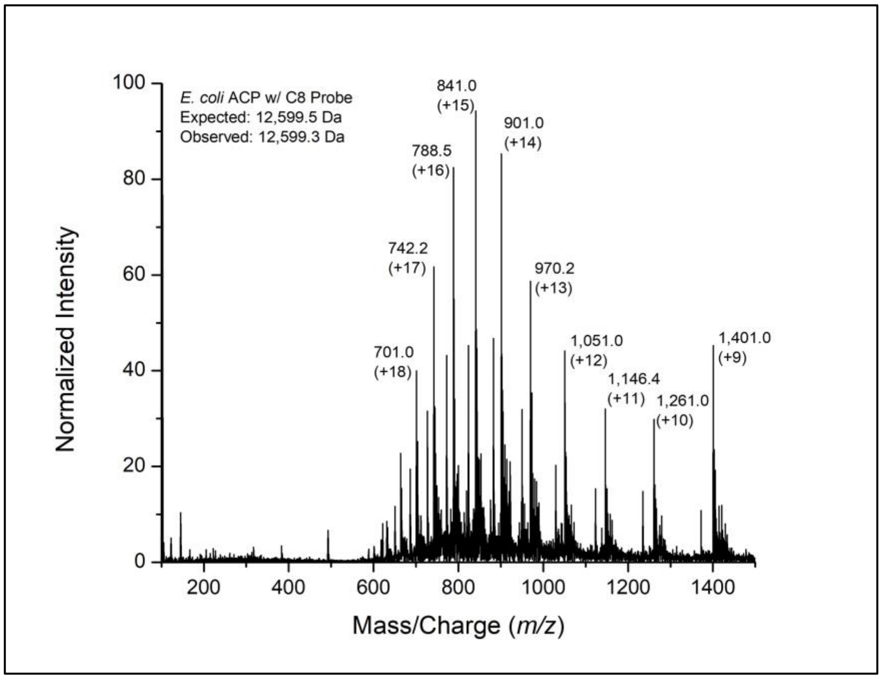

Supplementary Figure 11: ES mass spectrum of *acyl*-EcACP C8.
The different charge (z) states are marked on the plot and are calculated according to the theoretical molecular weight of the ACP (minus the N-terminal methionine).

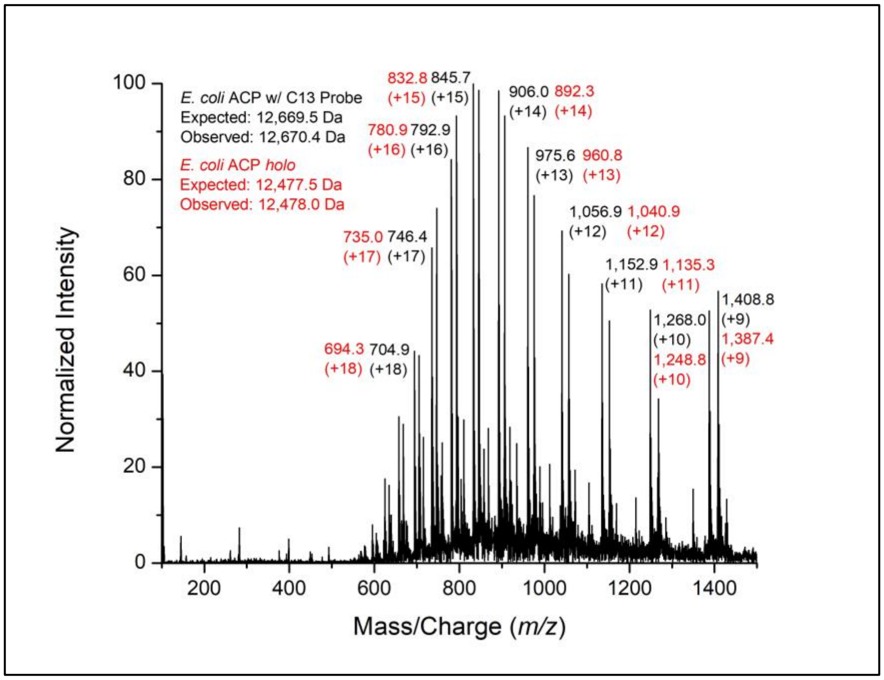

Supplementary Figure 12: ES mass spectrum of *acyl*-EcACP C13.
The different charge (z) states are marked on the plot and are calculated according to the theoretical molecular weight of the ACP (minus the N-terminal methionine).

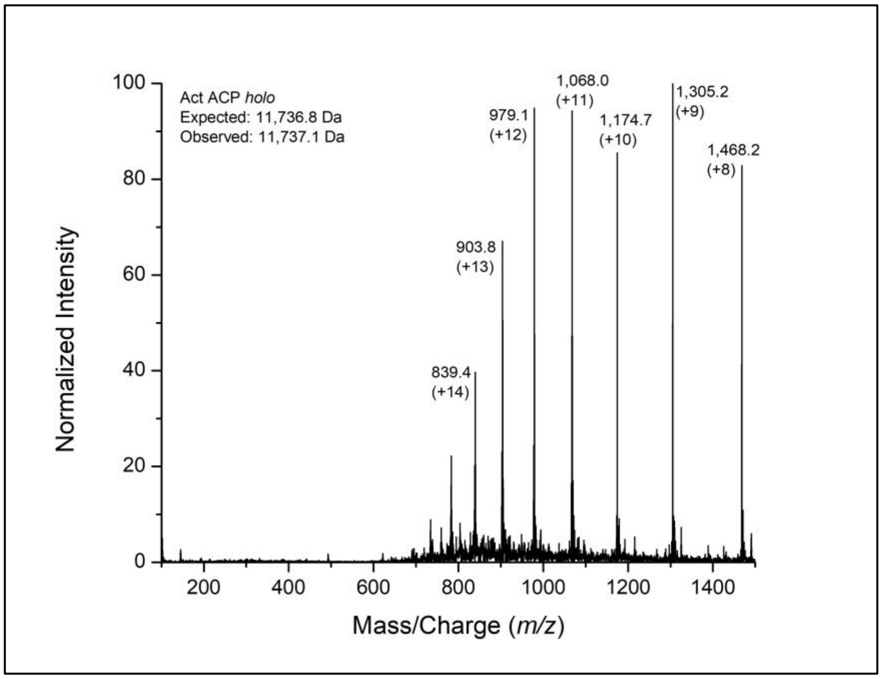

Supplementary Figure 13: ES mass spectrum of *holo*-Act ACP.
The different charge (z) states are marked on the plot and are calculated according to the theoretical molecular weight of the ACP (minus the N-terminal methionine).

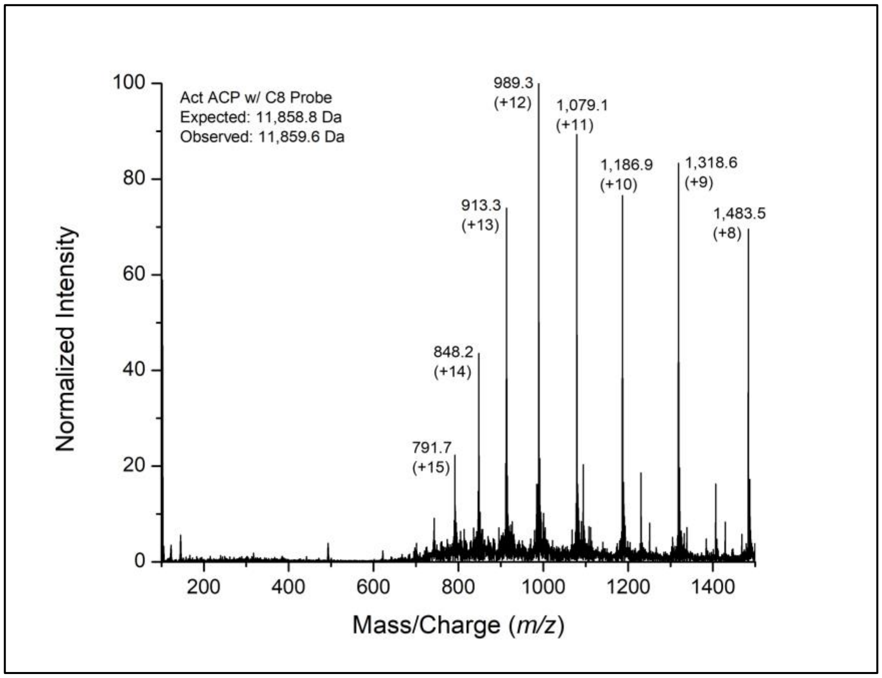

Supplementary Figure 14: ES mass spectrum of *acyl*-Act ACP C8.
The different charge (z) states are marked on the plot and are calculated according to the theoretical molecular weight of the ACP (minus the N-terminal methionine).

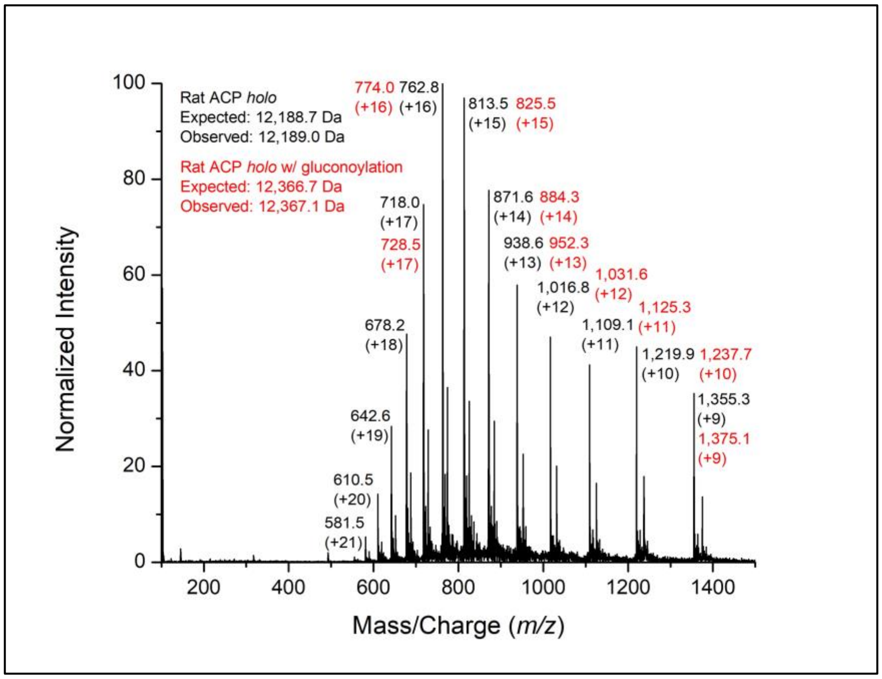

Supplementary Figure 15: ES mass spectrum of *holo*-Rat ACP.
The different charge (z) states are marked on the plot and are calculated according to the theoretical molecular weight of the ACP (minus the N-terminal methionine).

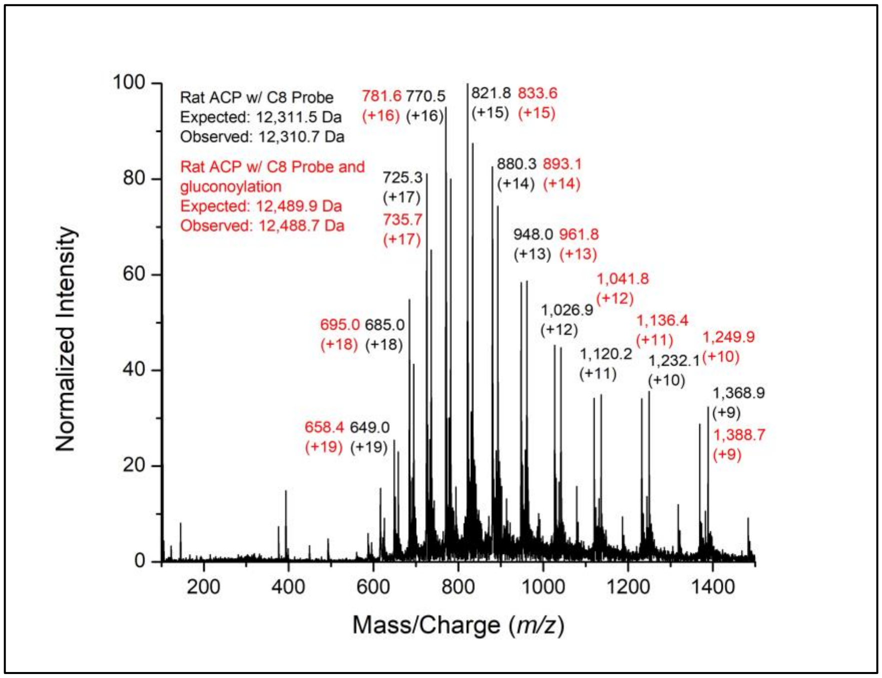

Supplementary Figure 16: ES mass spectrum of *acyl*-Rat ACP C8.
The different charge (z) states are marked on the plot and are calculated according to the theoretical molecular weight of the ACP (minus the N-terminal methionine).

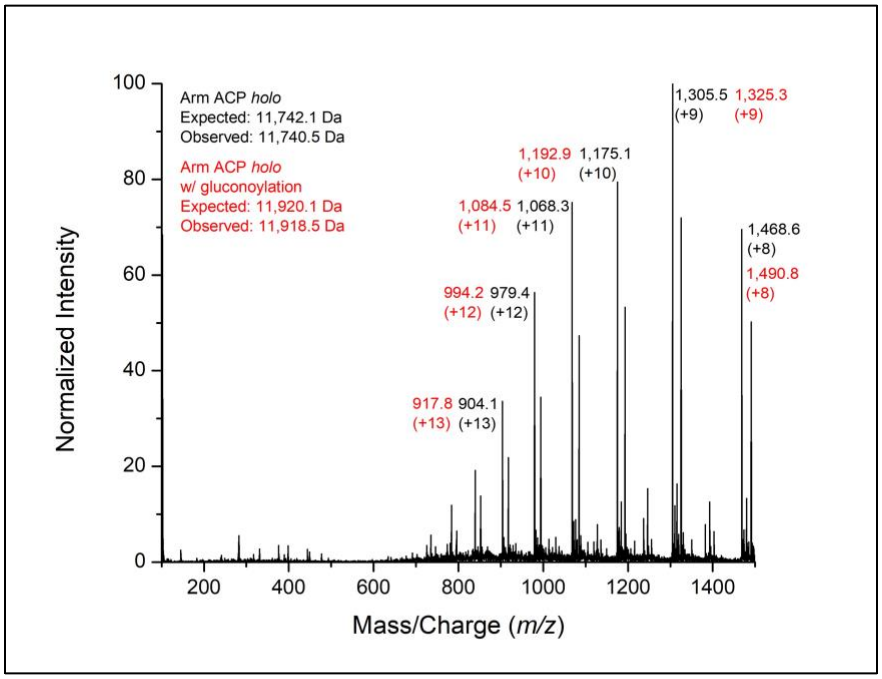

Supplementary Figure 17: ES mass spectrum of *holo*-Arm ACP.
The different charge (z) states are marked on the plot and are calculated according to the theoretical molecular weight of the ACP (minus the N-terminal methionine).

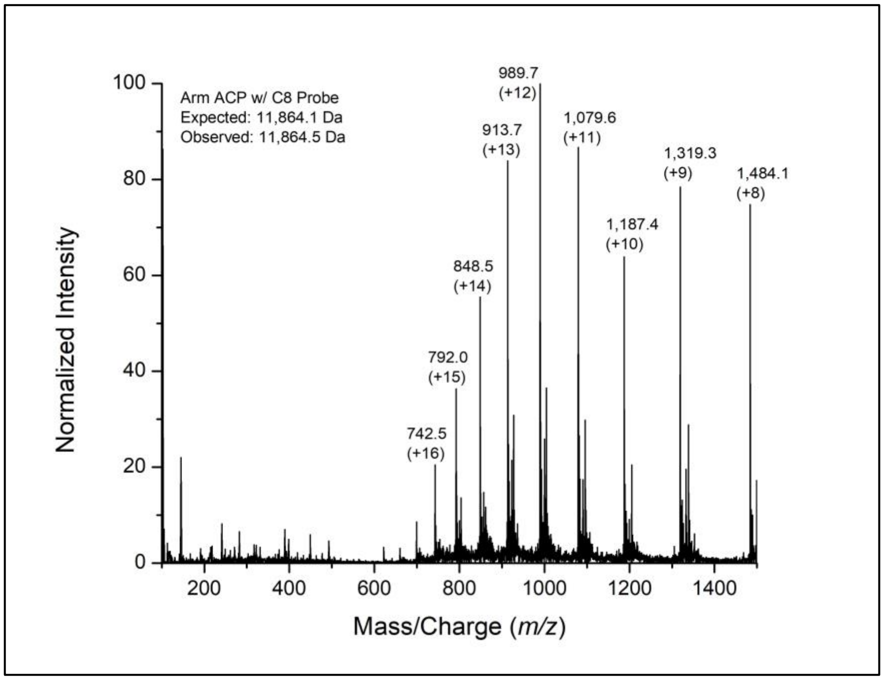

Supplementary Figure 18: ES mass spectrum of *acyl*-Arm ACP C8.
The different charge (z) states are marked on the plot and are calculated according to the theoretical molecular weight of the ACP (minus the N-terminal methionine).

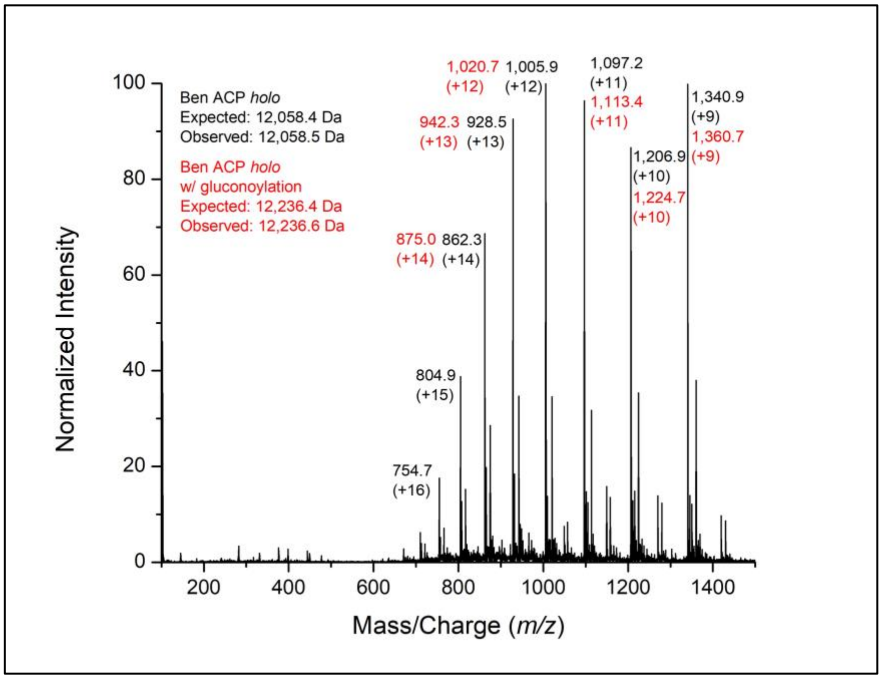

Supplementary Figure 19: ES mass spectrum of *holo*-Ben ACP.
The different charge (z) states are marked on the plot and are calculated according to the theoretical molecular weight of the ACP (minus the N-terminal methionine).

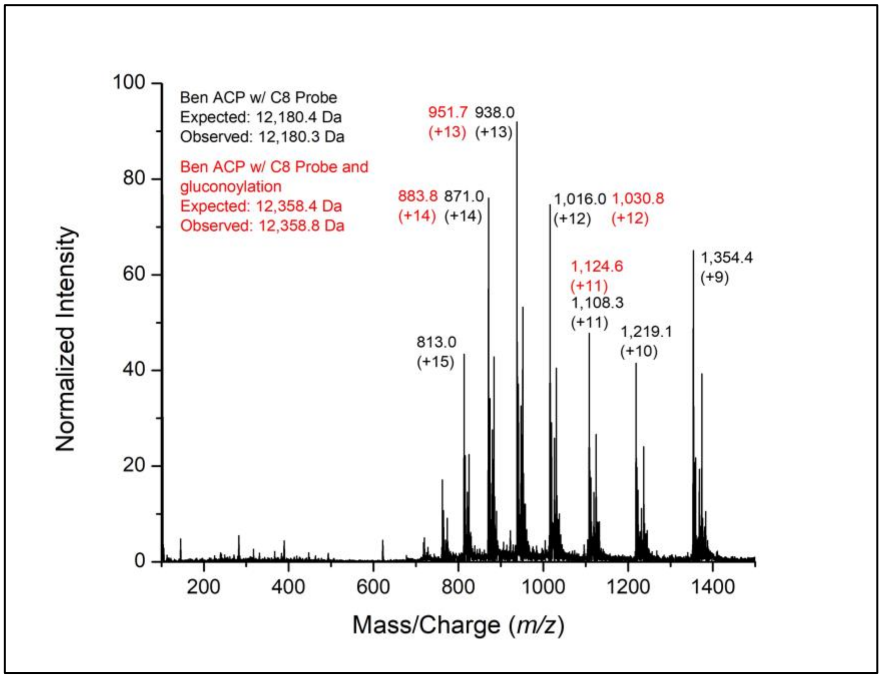

Supplementary Figure 20: ES mass spectrum of *acyl*-Ben ACP C8.
The different charge (z) states are marked on the plot and are calculated according to the theoretical molecular weight of the ACP (minus the N-terminal methionine).

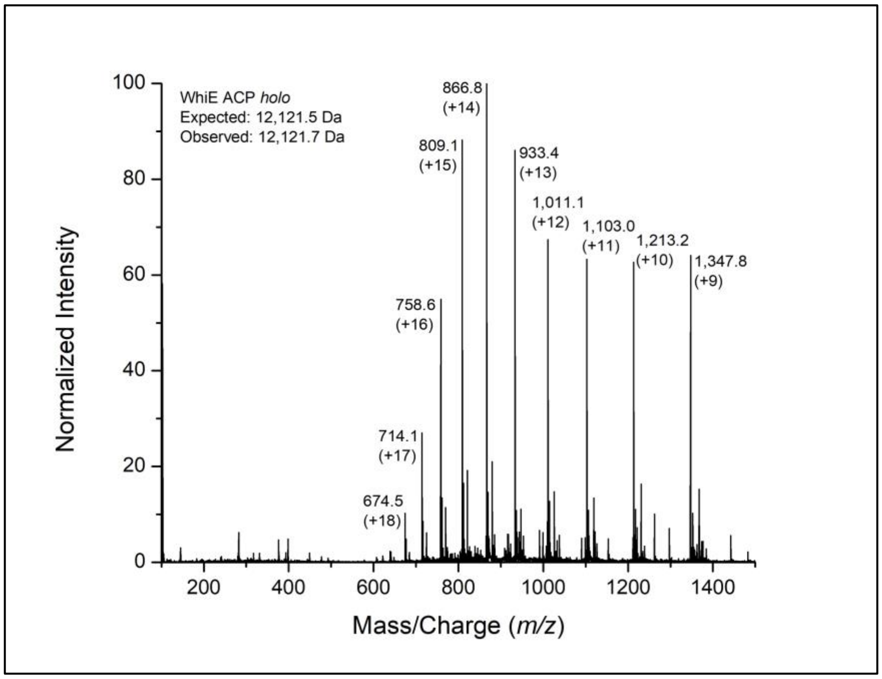

Supplementary Figure 21: ES mass spectrum of *holo*-WhiE ACP.
The different charge (z) states are marked on the plot and are calculated according to the theoretical molecular weight of the ACP (minus the N-terminal methionine).

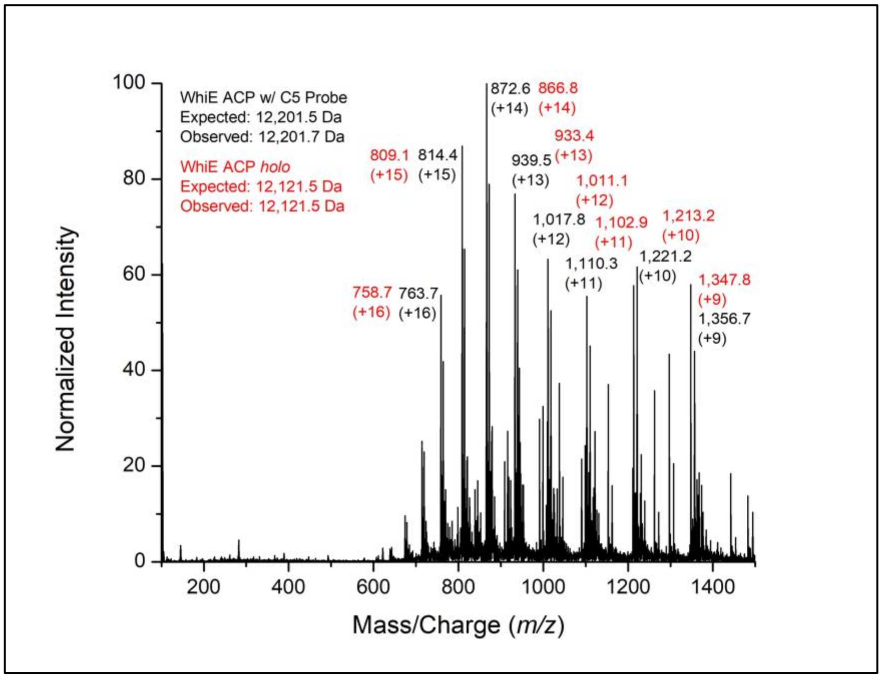

Supplementary Figure 22: ES mass spectrum of *acyl*-WhiE ACP C5.
The different charge (z) states are marked on the plot and are calculated according to the theoretical molecular weight of the ACP (minus the N-terminal methionine).

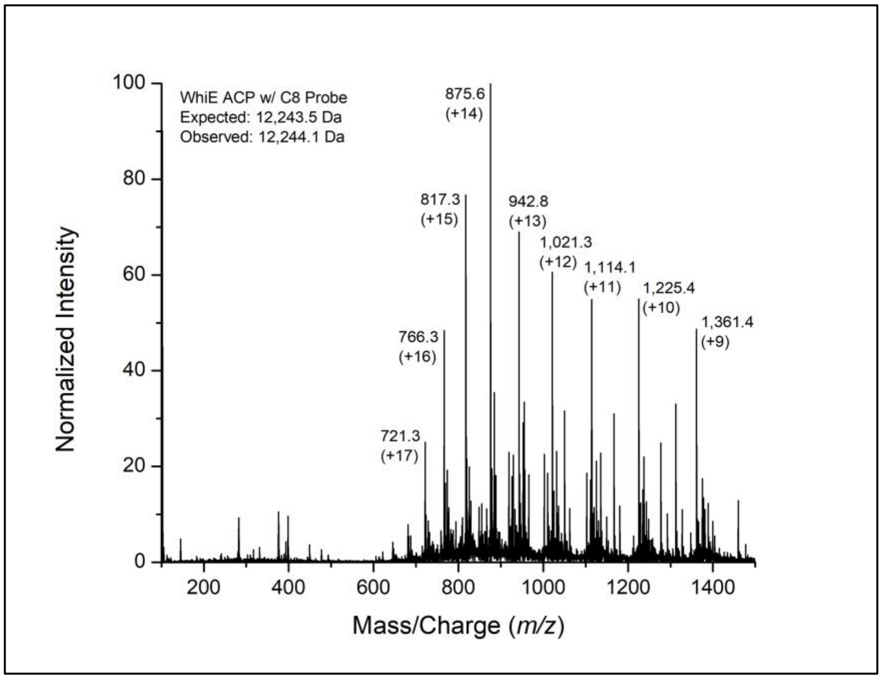

Supplementary Figure 23: ES mass spectrum of *acyl*-WhiE ACP C8.
The different charge (z) states are marked on the plot and are calculated according to the theoretical molecular weight of the ACP (minus the N-terminal methionine).

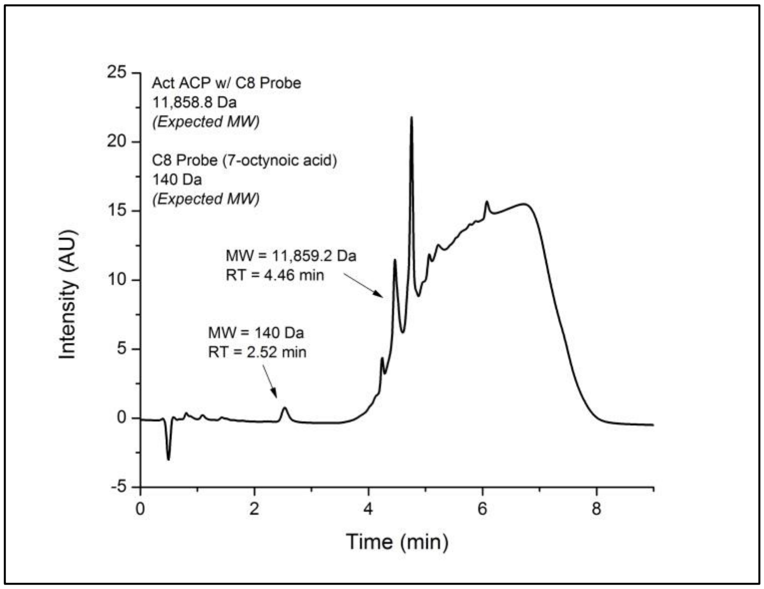

Supplementary Figure 24: LC trace from acyl-Act ACP C8 spiked with free probe.
It is important to ensure that the purification of the acyl-ACP after the AasS ligation reaction was effective at removing excess probe that remained in solution, unattached to the ACP. The presence of excess probe in addition to the probed-loaded ACP would produce the same Raman signal as a probe that is loaded onto the ACP and is solvent-exposed or not sequestered. Therefore, spiking experiments were performed to ensure that the presence of excess probe in solution would be detectable by LC-MS. The LC trace (measured at 254 nm) features *acyl*-Act ACP C8 (2.5 mM), spiked with the C8 Probe (250 μM). Distinct retention times are denoted for each species.

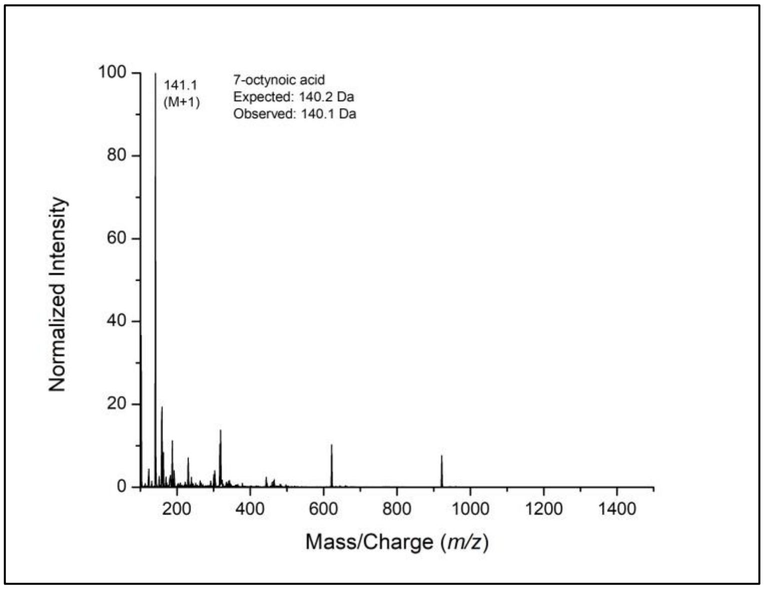

Supplementary Figure 25: ES mass spectrum of 7-octynoic acid (C8 probe).
A peak corresponding to the C8 Probe (see Supplementary Figure 24) is present at a retention time of 2.52 minutes in the LC Trace. The expected and observed molecular weight for 7-octynoic acid is 140 Da. These results suggest that at molar quantities of even 10% of the ACP, excess free probe would have been detected by LC-MS.

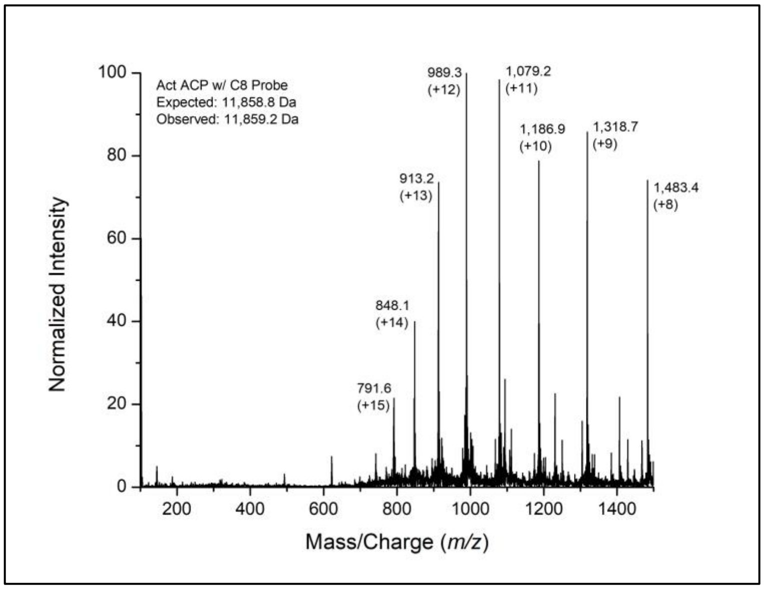

Supplementary Figure 26: ES mass spectrum of *acyl*-Act ACP C8.
A peak corresponding to the *acyl*-Act ACP C8 (see Supplementary Figure 24) is present at a retention time of 4.46 minutes in the LC Trace. The expected molecular weight for *acyl*-Act ACP C8 is 11,858.8 Da and the observed molecular weight is 11,859.2 Da.

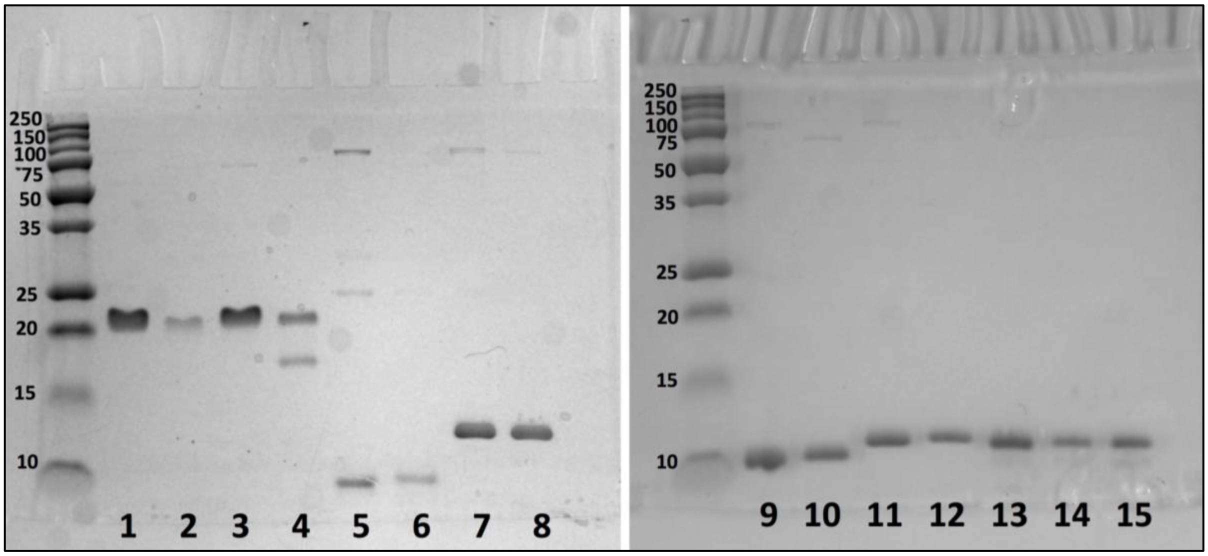

Supplementary Figure 27: SDS PAGE of *holo*- and *acyl*-ACPs.
SDS PAGE is used to compare protein samples with respect to size. Notably, this method can be used to differentiate ACPs from different biological systems. Protein ladder features labeled bands ranging from 10 kDa to 250 kDa. Lane 1: *holo*-EcACP; Lane 2: *acyl*-EcACP C5; Lane 3: *acyl*-EcACP C8; Lane 4: *acyl*-EcACP C13 (50% converted from *holo*); Lane 5: *holo*-Act ACP; Lane 6: *acyl*-Act ACP C8; Lane 7: *holo*-Rat ACP; Lane 8: *acyl*-Rat ACP C8; Lane 9: *holo*-Arm ACP; Lane 10: *acyl*-Arm ACP C8; Lane 11: *holo*-Ben ACP; Lane 12: *acyl*-Ben ACP C8; Lane 13: *holo*-WhiE ACP; Lane 14: *acyl*-WhiE ACP C5 (40% converted from *holo*); Lane 15: *acyl*-WhiE ACP C8. Source data are provided as a Source Data file.

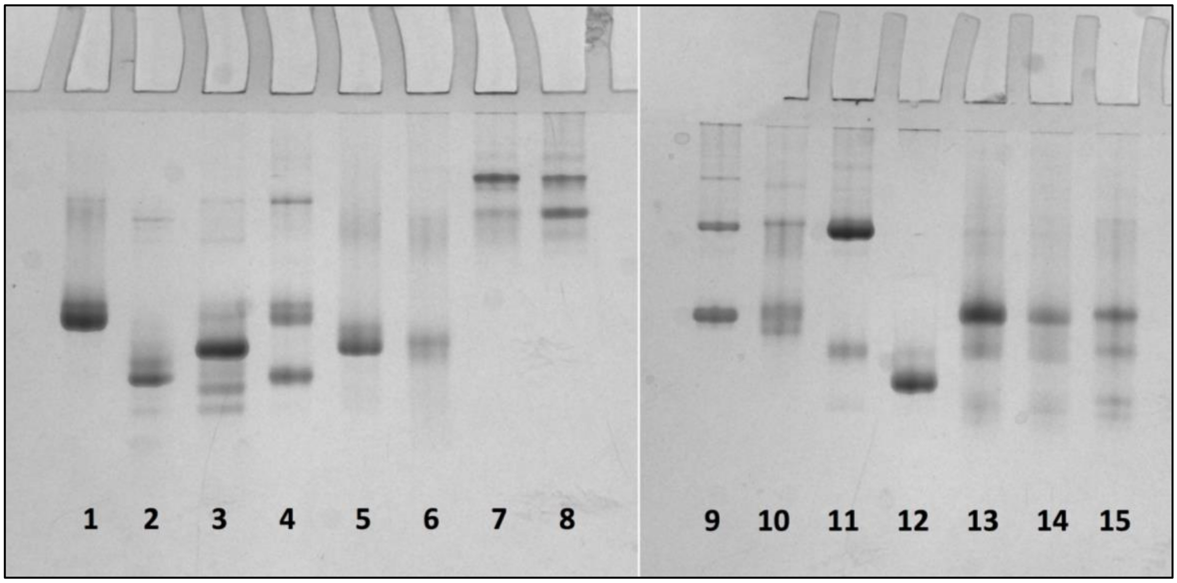

Supplementary Figure 28: Urea PAGE of *holo*- and *acyl*-ACPs.
Urea PAGE is used to qualitatively compare protein samples with respect to modification. Lane 1: *holo*-EcACP; Lane 2: *acyl*-EcACP C5; Lane 3: *acyl*-EcACP C8; Lane 4: *acyl*-EcACP C13 (50% converted from *holo*); Lane 5: *holo*-Act ACP; Lane 6: *acyl*-Act ACP C8; Lane 7: *holo*-Rat ACP; Lane 8: *acyl*-Rat ACP C8; Lane 9: *holo*-Arm ACP; Lane 10: *acyl*-Arm ACP C8; Lane 11: *holo*-Ben ACP; Lane 12: *acyl*-Ben ACP C8; Lane 13: *holo*-WhiE ACP; Lane 14: *acyl*-WhiE ACP C5 (40% converted from *holo*); Lane 15: *acyl*-WhiE ACP C8. Source data are provided as a Source Data file.

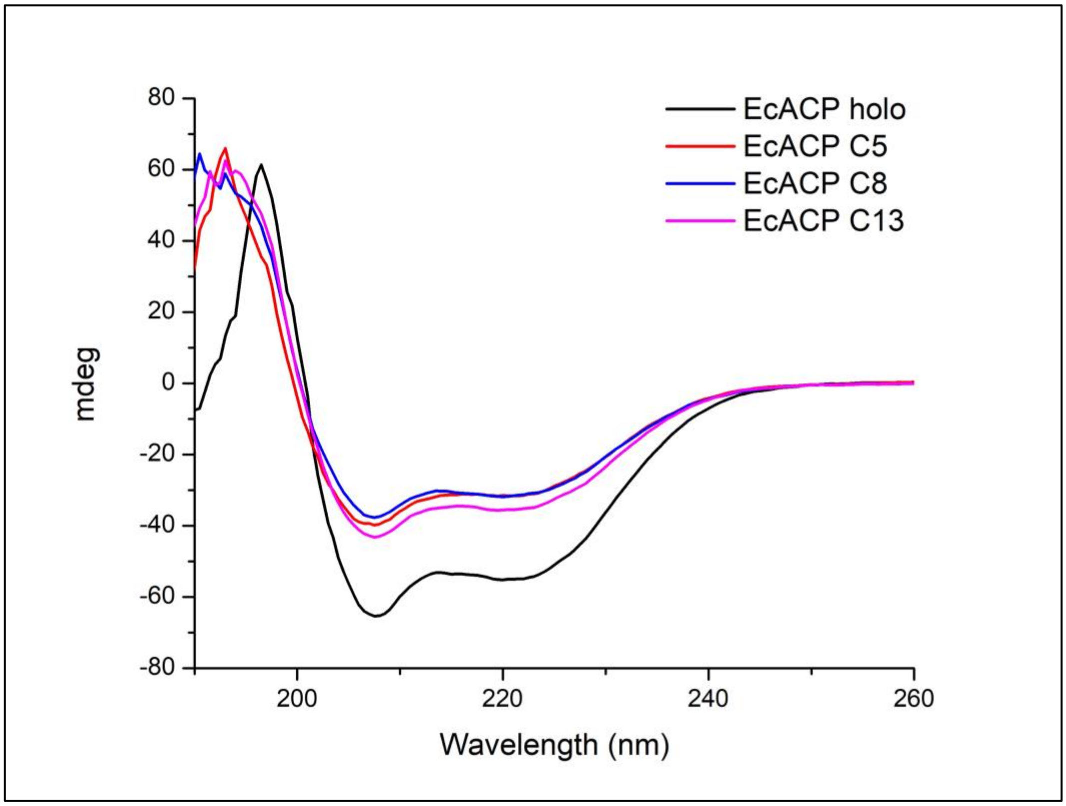

Supplementary Figure 29: CD spectra of EcACP with alkyne-labeled substrates.
CD spectra were acquired with and without the loading of alkyne-labeled substrates to ensure that the secondary alpha helical structure of the EcACP was maintained after the probe was loaded. The CD spectra shown above correspond to EcACP in the *holo* (unloaded Ppant arm) form as well as with various alkyne-labeled substrates (C5, C8, and C13 probes). As described in Methods, wavelength scans were collected at the 260–180 nm range on an Aviv model 410 spectropolarimeter. Characteristic peaks at 222 and 208 nm are indicative of the conservation of the secondary structure in ACPs featuring a labeled substrate. Source data are provided as a Source Data file.

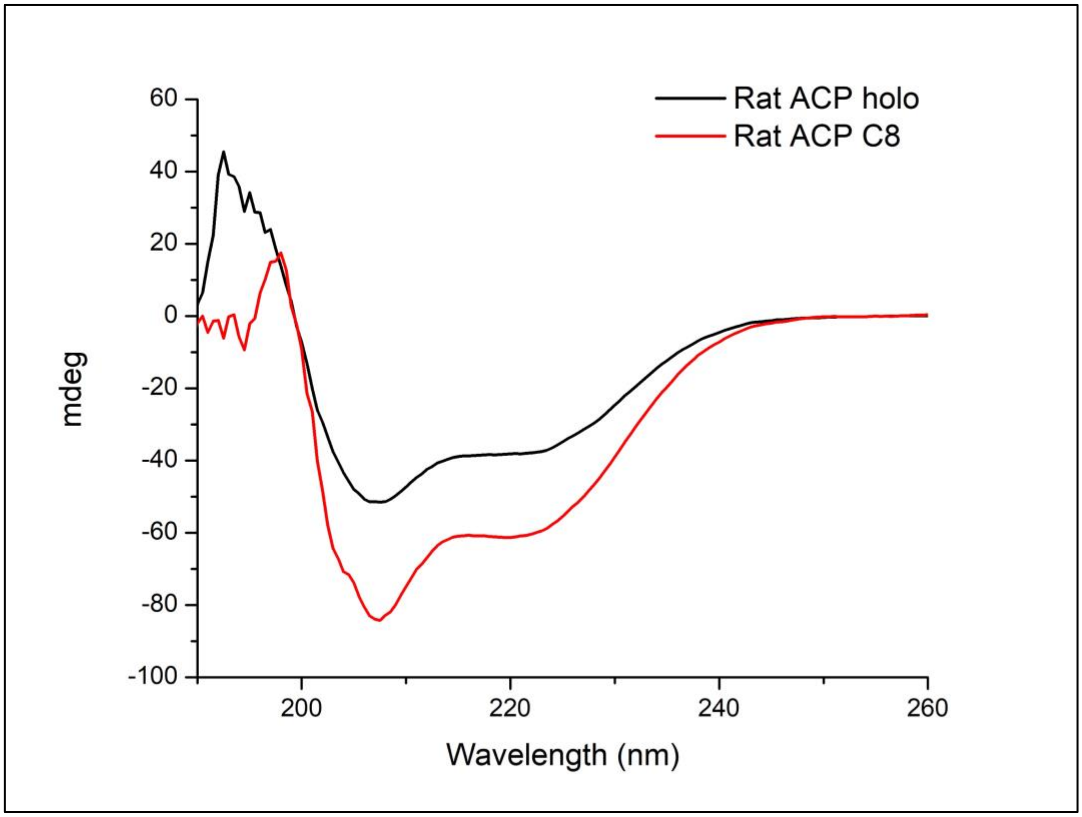

Supplementary Figure 30: CD spectra of Rat ACP with an alkyne-labeled substrate.
CD spectra were acquired with and without the loading of alkyne-labeled substrates to ensure that the secondary alpha helical structure of the Rat ACP was maintained after the probe was loaded. The CD spectra shown above correspond to Rat ACP in the *holo* (unloaded Ppant arm) form as well as with a C8 alkyne-labeled substrates. As described in Methods, wavelength scans were collected at the 260–180 nm range on an Aviv model 410 spectropolarimeter. Characteristic peaks at 222 and 208 nm are indicative of the conservation of the secondary structure in ACPs featuring a labeled substrate. Source data are provided as a Source Data file.

Supplementary Tables

Supplementary Table 1: Mode, mean, and FWHM of alkyne probe Raman peak.
For each Raman spectrum presented in this study, the mode, mean, and full width at half maximum (FWHM) of the alkyne peak are reported. For each protein spectrum, the mean and FWHM are compared to the spectrum of the respective unattached probe by reporting the difference.

Sample	Mode (cm^{-1})	Mean (cm^{-1})	FWHM (cm^{-1})	ΔMean Relative to Probe (cm^{-1})	ΔFWHM Relative to Probe (cm^{-1})
C5 Probe	2117.9	2117.2	13.1	n/a	n/a
C8 Probe	2112.6	2110.6	12.3	n/a	n/a
C13 Probe	2110.7	2111.1	11.6	n/a	n/a
EcACP C5	2117.9	2117.7	14.1	0.5	1.0
EcACP C8	2116.1	2115.2	12.7	4.6	0.4
EcACP C13	2116.0	2118.1	12.3	7.0	0.7
Act ACP C8	2112.6	2112.9	15.1	2.3	2.7
Rat ACP C8	2112.6	2110.7	12.7	0.1	0.3
Arm ACP C8	2112.6	2112.4	14.5	1.3	2.1
Ben ACP C8	2110.9	2110.6	14.8	0.1	2.5
WhiE ACP C8	2112.6	2111.2	12.7	0.6	0.4
WhiE ACP C5	2117.9	2118.2	12.8	1.0	-0.3

Supplementary References

1. W. Anderson, P. A mathematical model for the narrowing of spectral lines by exchange or motion. *J. Phys. Soc. Japan* **9,** 316–339 (1954).
2. Kubo, R. in *Fluctuation, Relaxation, and Resonance in Magnetic Systems* (ed. Ter Haar, D.) (Oliver and Boyd, 1962).
