# Supplementary material for: Tracking carrier protein motions with Raman spectroscopy
Source: Nat Commun. 2019 May 20;10:2227. doi: 10.1038/s41467-019-10184-2 (PMC6527581; doi:10.1038/s41467-019-10184-2)
Supplement: Supplementary file 3 — Description of Additional Supplementary Files [file 41467_2019_10184_MOESM3_ESM.pdf]

Description of Additional Supplementary Files

File name: Supplementary Data 1

Description: EcACP and Act ACP PDB entries inform on chain sequestration
