# Supplementary material for: Tracking carrier protein motions with Raman spectroscopy
Source: Nat Commun. 2019 May 20;10:2227. doi: 10.1038/s41467-019-10184-2 (PMC6527581; doi:10.1038/s41467-019-10184-2)
Supplement: Supplementary file 4 — Supplementary Data 1 [file 41467_2019_10184_MOESM4_ESM.pdf]

Supplementary Data 1 for
Tracking carrier protein motions with Raman spectroscopy

Epstein et al.

Supplementary Data 1: EcACP and Act ACP PDB entries inform on chain sequestration. Key: 1. PDB file name, 2. Substrate, 3. Method of acquisition of structure (X-ray crystallography or NMR*), 4. Natural or non-pathway substrate. In all images, the ACP is colored (EcACP is green and Act ACP is blue), the substrate carbon chain is magenta, and the Ppant arm is colored by atom type. *The extreme state from the available ensemble of protein structures (most or least sequestered) was selected for use in this table.

EcACP (includes only structures that are able to resolve the Ppant arm**)
**Additional reported structures include: 2k92, 2k93, 2k94, 1L0H, and 1L0I, which do not resolve the Ppant arm or substrate.

Structure	Most Sequestered State	Least Sequestered State
1. 2FAC<br>2. Hexanoyl acyl chain<br>3. X-ray<br>4. Natural	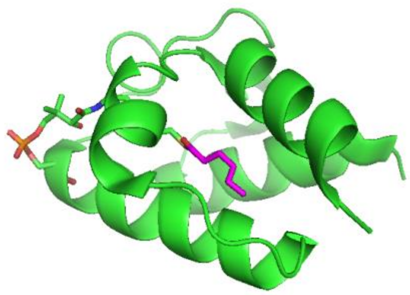	N/A
1. 2FAD<br>2. Heptanoyl acyl chain<br>3. X-ray<br>4. Natural	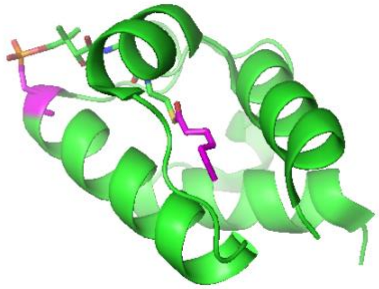	N/A
1. 2FAE<br>2. Decanoyl acyl chain<br>3. X-ray<br>4. Natural	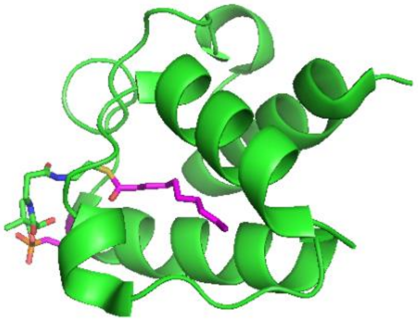	N/A

Act ACP

Structure	Most Sequestered State	Least Sequestered State
1. 2KG6<br>2. Acetyl acyl chain<br>3. NMR*<br>4. Non-pathway	N/A	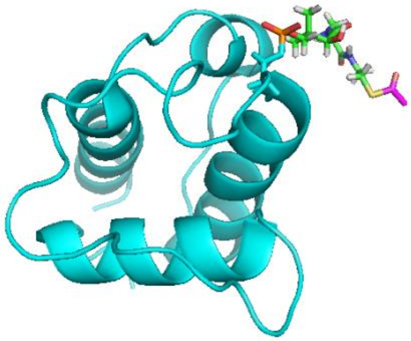
1. 2KG9<br>2. Butyryl acyl chain<br>3. NMR*<br>4. Non-pathway	N/A	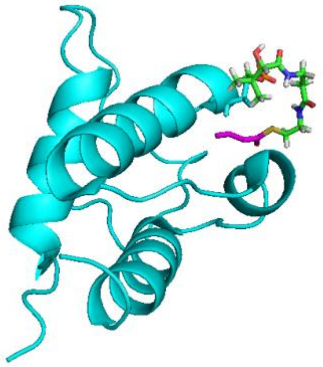
1. 2KGA<br>2. Hexanoyl acyl chain<br>3. NMR*<br>4. Non-pathway	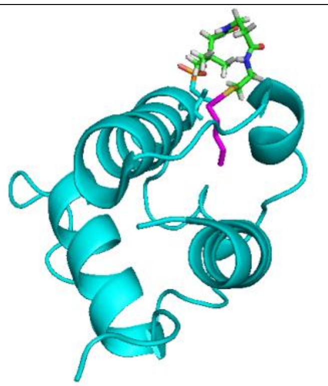	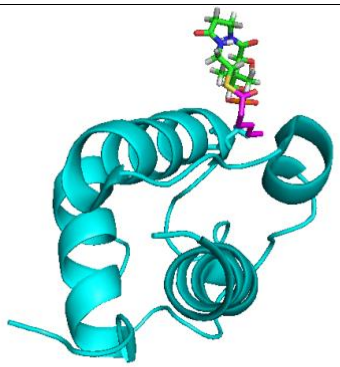

1. 2KGC<br>2. Octanoyl acyl chain<br>3. NMR*<br>4. Non-pathway	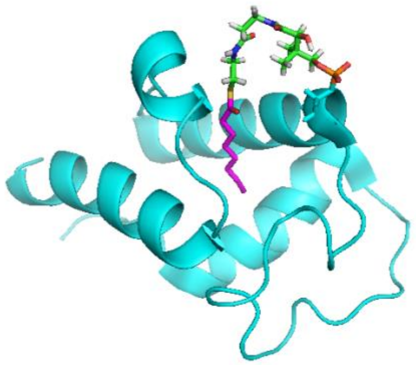	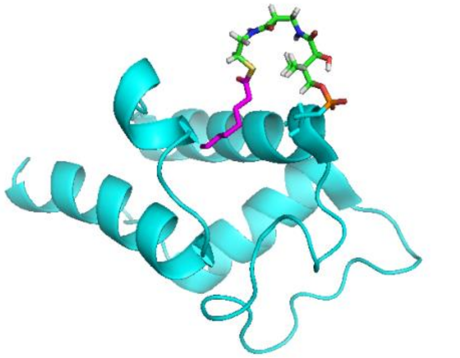
1. 2K0X<br>2. none (*holo*)<br>3. NMR*<br>4. n/a	N/A	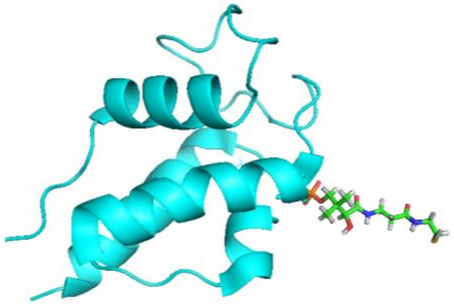
1. 2KG8<br>2. Malonyl group<br>3. NMR*<br>4. Natural	N/A	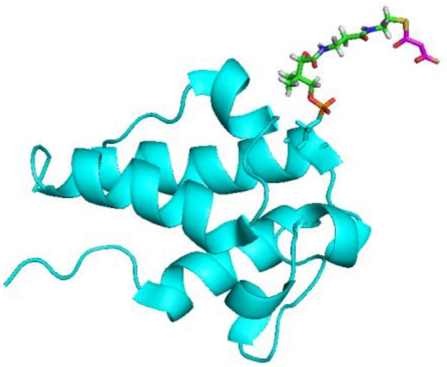
1. 2KGE<br>2. 3,5-dioxohexyl group<br>3. NMR*<br>4. Natural	N/A	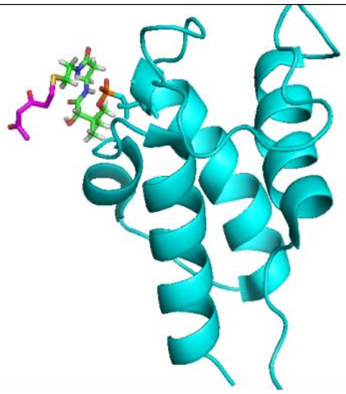

1. 2KGD<br>2. 3-oxo-butyl group<br>3. NMR*<br>4. Natural	N/A	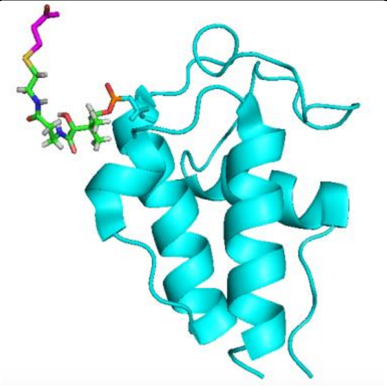
1. 2MVU<br>2. 3,7-dioxo-octyl group<br>3. NMR*<br>4. Natural	N/A	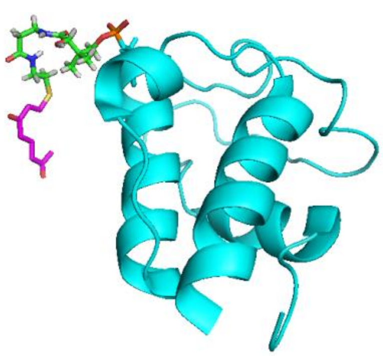
1. 2MVV<br>2. 5-phenyl-3-oxo-pentyl group<br>3. NMR*<br>4. Natural	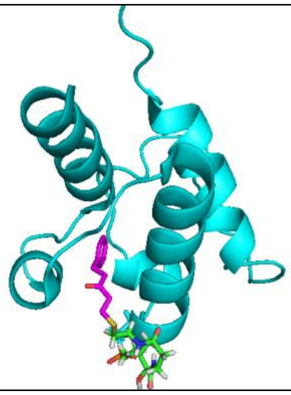	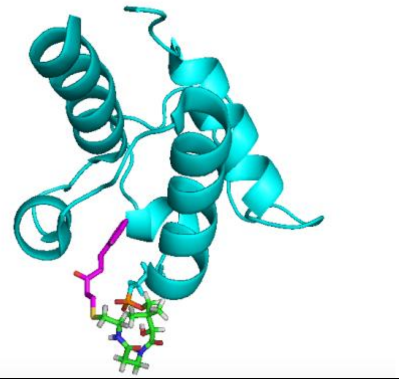
